# Summarizing and Communicating on Survival Data According to the Audience: A Tutorial on Different Measures Illustrated with Population-Based Cancer Registry Data [Corrigendum]

**DOI:** 10.2147/CLEP.S273165

**Published:** 2020-08-19

Belot A, Ndiaye A, Luque-Fernandez MA, et al. *Clin Epidemiol*. 2019;11:53–65.

Page 57, Number of life years lost (NLYL) section, left column, the sentence “where the quantity 1–*F_P_*(*t*) can be replaced by *S_P_*(*t*), ie, the classical survival function using the population mortality rates λ_P_” placed immediately after equation 14 indicates that the quantity 1–*F_P_*(*t*) could be replaced by *S_P_*(*t*). This is wrong as the one-to-one relationship between hazard and risk applies only in all-cause mortality setting but not in competing risks settings (such as the cause-specific or relative survival setting). Indeed, *F_P_*(*t*) depends on both hazards (cancer and other causes) through the overall survival S(*t*).

The authors therefore request the reader to ignore this sentence and apologize for this error.

